# Running barefoot leads to lower running stability compared to shod running - results from a randomized controlled study

**DOI:** 10.1038/s41598-021-83056-9

**Published:** 2021-02-23

**Authors:** Karsten Hollander, Daniel Hamacher, Astrid Zech

**Affiliations:** 1 Faculty of Medicine, MSH Medical School Hamburg, Am Kaiserkai 1, 20457 Hamburg, Germany; 2 Department of Sport Science, Friedrich Schiller University Jena, Jena, Germany

**Keywords:** Musculoskeletal system, Biophysics, Outcomes research, Clinical trial design

## Abstract

Local dynamic running stability is the ability of a dynamic system to compensate for small perturbations during running. While the immediate effects of footwear on running biomechanics are frequently investigated, no research has studied the long-term effects of barefoot vs. shod running on local dynamic running stability. In this randomized single-blinded controlled trial, young adults novice to barefoot running were randomly allocated to a barefoot or a cushioned footwear running group. Over an 8-week-period, both groups performed a weekly 15-min treadmill running intervention in the allocated condition at 70% of their VO_2_ max velocity. During each session, an inertial measurement unit on the tibia recorded kinematic data (angular velocity) which was used to determine the short-time largest Lyapunov exponents as a measure of local dynamic running stability. One hundred running gait cycles at the beginning, middle, and end of each running session were analysed using one mixed linear multilevel random intercept model. Of the 41 included participants (48.8% females), 37 completed the study (drop-out = 9.7%). Participants in the barefoot running group exhibited lower running stability than in the shod running group (*p* = 0.037) with no changes during the intervention period (*p* = 0.997). Within a single session, running stability decreased over the course of the 15-min run (*p* = 0.012) without differences between both groups (*p* = 0.060). Changing from shod to barefoot running reduces running stability not only in the acute phase but also in the longer term. While running stability is a relatively new concept, it enables further insight into the biomechanical influence of footwear.

## Introduction

Barefoot vs. shod running has achieved an increased public and scientific attention over the last years with advocates on both sides of the cushioning spectrum^1–4^. Studies focussing on barefoot running primarily investigate biomechanical, physiological or injury-related outcomes and a final conclusion on its benefits or detriments still needs to be determined^5^.

While the dependence of various biomechanical variables on footwear and their implications on injuries has been frequently investigated over the last decades^6–10^, nonlinear running gait features have rarely been addressed^11–13^. On the basis of nonlinear time series analysis, the local dynamic stability or running stability can be calculated^11,12,14^. In the current literature, this was done based on the vertical trunk coordinates^11,14^ as well as on the angular velocity data of the thorax, pelvis or foot^12^. Small internal or external perturbations are present during each movement and affect the neuromuscular and locomotion system^15^. To quantify, how well the locomotion system responds to these small perturbations during walking and running, the largest Lyapunov exponent can be used^15,16^ as a measure of local dynamic running stability (also known as local dynamic stability; LDS). Local dynamic running stability is defined as the ability of a dynamic system to compensate for small perturbations during running. While for walking a low capacity of compensating for small perturbations (reduction of LDS) can directly be translated into clinical relevance (increased risk of falls)^15,17^, for running the direct causal link is still missing. However, a lower running stability with the associated lower capacity of compensation for small perturbations may increase the risk for overuse injuries such as bone stress injuries resulting from repetitive monotonous loads that exceed bone loading capacity^18,19^.

Running stability can be affected by running surface, footwear (stability based on the vertical trunk displacement)^11,14^, running experience (stability based on the angular velocity of the foot) and fatigue (stability based on the angular velocity of the pelvis and trunk)^12^. It has also been shown that the running stability increases during an exertional 5000 m run (measured at the beginning, the middle and the end of the run, stability based on the angular velocity of the pelvis) and is higher in elite runners compared to recreational runners (stability based on the angular velocity of the foot)^12^. Furthermore, Ekizos, et al.^11^ found a decreased running stability (based on the vertical trunk displacement) when habitually shod runners change to barefoot running. This decreased running stability was accompanied by the same changes of running gait parameters seen in other studies, such as a more anterior foot strike pattern, increased cadence, decreased step length and reduced contact time^11,20,21^. In another study, decreased running stability was induced after changing from a rearfoot strike pattern to a more anterior strike pattern in the short term^22^. However, the differences were alleviated after a 14-week transition to the more anterior footstrike pattern and, thus, discussed to be a reaction of the locomotor system to a new running strategy^22^. While the specific physiological mechanisms that influence running stability are unclear, it can be assumed that running barefoot challenges the sensorimotor system in habitual shod runners^11,23,24^.

While many studies investigated acute effects of barefoot vs. shod running, some recent studies investigated effects of barefoot vs. shod running on biomechanics over time^7,25,26^. However, the effect of repeated test conditions period of running barefoot vs. shod running on running stability has not been investigated.

Therefore, this study aimed to determine the effects of repeated test conditions to barefoot running on nonlinear biomechanics, measured as running stability. The running stability is expressed by the largest Lyapunov exponent based on the angular velocity of the tibia. We hypothesized that a new unfamiliar situation of barefoot running (compared to shod running) would show a decreased running stability in the beginning and that this difference would alleviate over seven sessions in an 8-week period.

## Methods

### Study design

This study was part of a randomized single-blinded controlled study with an intervention lasting 8 weeks. Results from this study regarding the overall outcomes have already been reported elsewhere^27,28^ and this study analysed weekly measurements during the individual intervention sessions. The reporting of this study adhered to the CONSORT (Consolidated Standards of Reporting Trials) statement^29^ and it was registered in the German Clinical Trial Register (DRKS00011073, date of registration: 11/01/2017). This study was approved by the institutional review board of the University of Hamburg (protocol number ID37) and all research was performed in accordance with relevant guidelines/regulations. Informed written consent was obtained from all participants.

### Participants and setting

Physically active and habitually shod participants between 18 and 35 years of age were recruited from the university surrounding (Table 1). Participants were novice to barefoot running and no specific running experience was required. Further exclusion criteria were a habituation to any barefoot or minimally shod sports (e.g. barefoot running, beach volleyball, taekwondo, karate, ballet, gymnastics) and any injury or neuromuscular disease in the six months prior to study. The recruitment and conduct took place between April 2016—April 2017 in the university biomechanics laboratories.

**Table 1:** Mean ± standard deviation of participant demographics.

	Barefoot intervention group (n = 21)	Footwear intervention group (n = 20)
Age [years]	25.2 ± 3.4	25.2 ± 2.9
Height [cm]	175.3 ± 7.6	177.7 ± 8.3
Weight [kg]	71.3 ± 12.2	71.4 ± 10.8
BMI [kg/m^2^]	23.0 ± 2.5	22.5 ± 1.9
Sex (percentage females)	52.4%	50.0%

### Interventions and randomisation

Block randomization was performed by the same research team member with stratification for sex. Participants were assigned to a barefoot or a shod intervention group. There was a third passive group that did not perform any running intervention^28^. Since such a passive group is not relevant for the analysis of the hypothesis, the data of this group was not analysed in this study.

The researcher (DH) involved in the data processing and statistical analysis was blinded to study arm allocation. Participants and researchers administering the treatment were not able to be blinded.

### Intervention

After conducting a VO_2_max test on a treadmill (Quark CPET COSMED, Rome, Italy)^30^ in week one, participants received seven intervention sessions in the allocated footwear condition. The seven sessions were one week (± 1 day) apart from each other and consisted of 15 min of running on an instrumented treadmill at 70% of their individual VO_2_max velocity to prevent exhaustive effects (TRAC 4000, Ergo-Fit GmbH & Co. KG, Pirmasens, Germany). Participants were allowed to miss a maximum of one session to be included in the final analysis.

For the shod intervention, a new cushioned running shoe (Asics Cumulus 17, 10 mm heel drop, neutral arch support, weight: 336 g for US size male 9) was individually used for every participant and stayed in the laboratory during the conduct of the study. The barefoot intervention was conducted barefoot. Participants were allowed to continue their physical activity/sports in their usual footwear but were requested to not initiate any barefoot sport.

### Instrumentation

To register running gait kinematics, an inertial measurement unit (IMU, Shimmer3, Shimmer, Dublin, Ireland; gyroscope range of measurement: ± 2000°/s; sampling rate: 256 Hz) was fixed to the tibia (medial and distal to the tibial tuberosity) with an elastic strap. The measurement was started while the participants were standing on the treadmill.

### Data processing

The unfiltered IMU data (three-dimensional angular velocity) was exported using the software Consensys (version 1.6, Shimmer, Dublin, Ireland). The following data processing was conducted with self-made MATLAB (version 2014a; The MathWorks, Inc., Natick, USA) scripts. Since the measurement was started and stopped while the participant stood on the treadmill, the start and end of the running sessions were visually detected.

### Primary outcome

As a prerequisite for calculating LDS foot–ground contacts were identified as local minima of the angular velocity of the sagittal plane as described previously for human gait^31^. Thereafter, the first and last 50 running gait cycles were excluded from the following data analysis.

The primary outcome of this study was the running stability measured as the LDS. LDS has frequently been used to assess non-linear walking biomechanics and less frequently been applied to running biomechanics^11,12,22,32^. We calculated the LDS based on the angular velocity data of the tibia from the first, middle and last 100 running gait cycles of each session. The method to determine LDS has been described extensively^33,34^. In brief, the three-dimensional angular velocity data of each 100 consecutive running gait cycles were time-normalized to 10,000 samples^35^. To reconstruct a state space, we applied the time-delayed embedding method. The time delay (τ) was chosen based on the first minimum mutual information analysis^36^ which was determined for each plane separately (x-axis [mean ± standard deviation]: τ = 7.6 ± 1.7; y-axis: τ = 12.2 ± 2.4; z-axis: τ = 11.8 ± 2.2). The mean (τ = 11) was used as a fixed time delay in each state space reconstruction for all participants. The embedding dimension (dE) was determined using the global false nearest neighbour analysis^37^. False neighbors were identified by comparing the actual state space with a state space including an additional time delayed copy of the 3D kinematic data (R_tol_ = 15.0 and A_tol_ = 2.0). Therefore, dE could only be a multiple of 3 (e.g. dE = 3, dE = 6, dE = 9 etc.). Since the attractor formed using delayed reconstruction is equivalent to the attractor in the unknown space of the original system only if the embedding dimension is sufficiently large, we used the maximum dE across all participants as the fixed embedding dimension (maximum dE = 9, mean dE = 6.2, standard deviation of the dE = 0.3)^38^. Taken together, the following state space ‘S(t)’ was reconstructed:

$${{\rm S}}\left( {{\rm t}} \right) = [{{\rm a{_{\rm x}}}}\left( {{\rm t}} \right),{{\rm a{_{\rm y}}}}\left( {{\rm t}} \right),{{\rm a{_{\rm z}}}}\left( {{\rm t}} \right),{{\rm a{_{\rm x}}}}({{\rm t}} +\uptau ),{{\rm a{_{\rm y}}}}({{\rm t}} +\uptau ),{{\rm a{_{\rm z}}}}({{\rm t}} +\uptau ),{{\rm a{_{\rm x}}}}({{\rm t}} + 2\uptau ),{{\rm a{_{\rm y}}}}({{\rm t}} + 2\uptau ),{{\rm a{_{\rm z}}}}({{\rm t}} + 2\uptau )],$$

with ‘a’ representing the angular velocity data (the subscript indicates the corresponding sensor axis) and τ the time delay (in our case 11).

**Figure 1:**
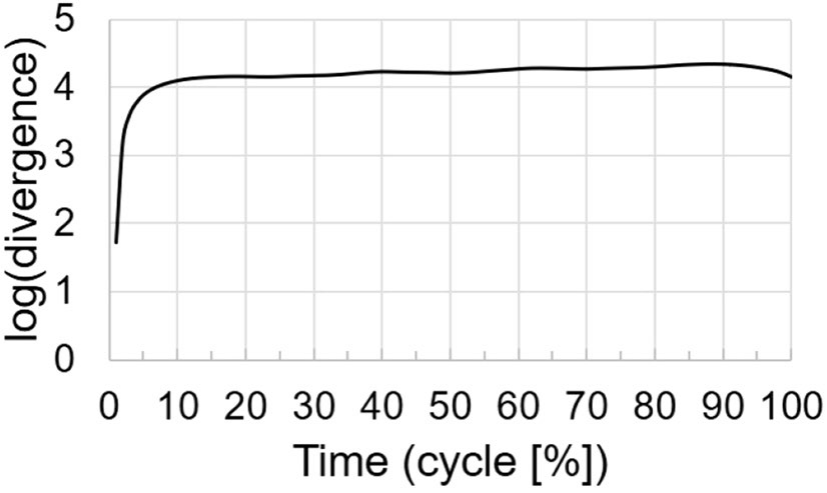
Divergence curve (mean across participants of the middle of the 15-min sessions)

The short-time largest Lyapunov exponent was calculated upon the state space using the algorithm of Rosenstein, et al.^39^. Thereto, for each point in the state space, we searched for the nearest neighbour (Euclidean distance) and tracked the distance of the initially nearest neighbour as it evolves in time. The short-time largest Lyapunov exponent is then defined as the slope of the linear fit of the mean of the logarithm of these divergence curves. Based on a visual inspection (Fig. 1) of the mean of the logarithm of this divergence curves, we fitted the line to a time frame representing the first 5% (5 time-normalised samples) of the running gait cycle.

### Statistical methods

We used a linear multi-level random intercept model (level 1: measurement within each participant; level 2: participants) to analyse the fixed effects group (barefoot vs. shod running), intersession time (the 7 sessions), and intrasession time (the beginning, middle and end of each session) on LDS (the largest Lyapunov exponent). Furthermore, all possible interaction effects were included into the model. The model was analysed using the restricted maximum likelihood (REML) estimator in IBM SPSS Statistics for Windows (Version 22.0. Armonk, NY: IBM Corp). Differences between groups were compared with independent t-tests (age and BMI) and Fisher’s excact χ^2^ test. The significance level was set to α = 5%.

## Results

### Participants

**Figure 2:**
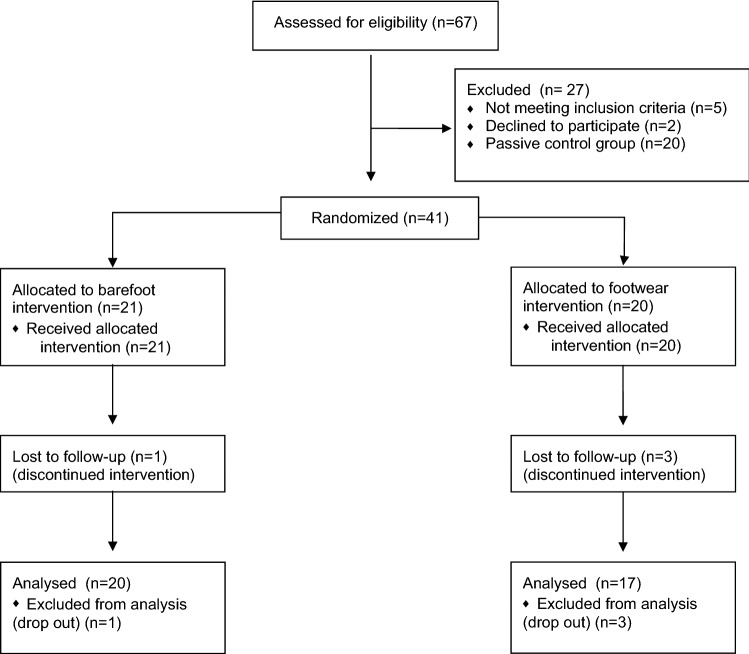
Flowchart of participants.

Overall, 41 participants were included in this randomised controlled trial (48.8% females, mean ± SD age 25.2 ± 3.1 years, BMI 22.8 ± 2.2 kg/m^2^) with no statistically significant differences between groups for age (*p* = 0.992), BMI (*p* = 0.424) or sex (*p* = 1.000) (Table 1). Three participants discontinued the intervention in the shod and one participant in the barefoot group (drop-out-rate: 9.7%). Reasons for the drop-outs were not related to the intervention (three illnesses and one anterior cruciate ligament rupture). The overall adherence with the training session was 97.3% (98.5% in the shod group and 96.3% in the barefoot group). The participant flow through the study can be found in Fig. 2. There were no adverse events during the conduct of the study.

### Decreased running stability during barefoot running compared to shod running

We found a significant group effect, participants in the barefoot running condition exhibited lower running stability compared to participants running shod (t_(group)_ = 2.1, *p* = 0.037, b = 1.924). This group effect was existent in the first session, over the course of the intervention and this difference remained unchanged (t_(group*__intersession time__)_ = −0.5, *p* = 0.633, b = −0.045). No effect of the intersession time was shown (t_(intersession time)_ < 0.1, *p* = 0.997, b < 0.001) (Table 2). Taken together, there was a significant group effect (barefoot vs. shod running) that remained unchanged within the session as well as across the sessions.

**Table 2:** Effects of group (shod n = 17; barefoot n = 20), intersession time (the 7 sessions), intrasession time (the beginning, middle, and end of each session), and all possible interaction effects on dynamic running stability (the largest Lyapunov exponent).

Fixed effects	b	t (df)	*p*	95% CI
Intercept	47.998	76.597 (52.746)	< 0.001	[46.741 to 49.255]
Intrasession time	0.579	2.521 (510.645)	0.012	[0.128 to 1.030]
Group (reference: shod = 0)	1.924	2.137 (52.840)	0.037	[0.118 to 3.729]
Intersession time	0.000	0.004 (512.140)	0.997	[−0.126 to 0.127]
Intrasession time * Group	−0.612	−1.881 (510.639)	0.060	[−1.252 to 0.027]
Intrasession time * Intersession time	−0.082	−1.680 (510.641)	0.094	[−0.178 to 0.014]
Group * Intersession time	−0.045	−0.478 (513.141)	0.633	[−0.228 to 0.139]
Intersession time * Group * Intrasession time	0.113	1.596 (510.637)	0.111	[−0.026 to 0.253]

Random effects	Estimates	Wald Z	*p*	95% CI
Residual	1.922	15.979	< 0.001	[1.700 to 2.173]
Intercept	5.385	3.924	< 0.001	[3.268 to 8.873]

The effects were analysed with a linear 2 level random intercept model.

### Running stability within a running session

**Figure 3:**
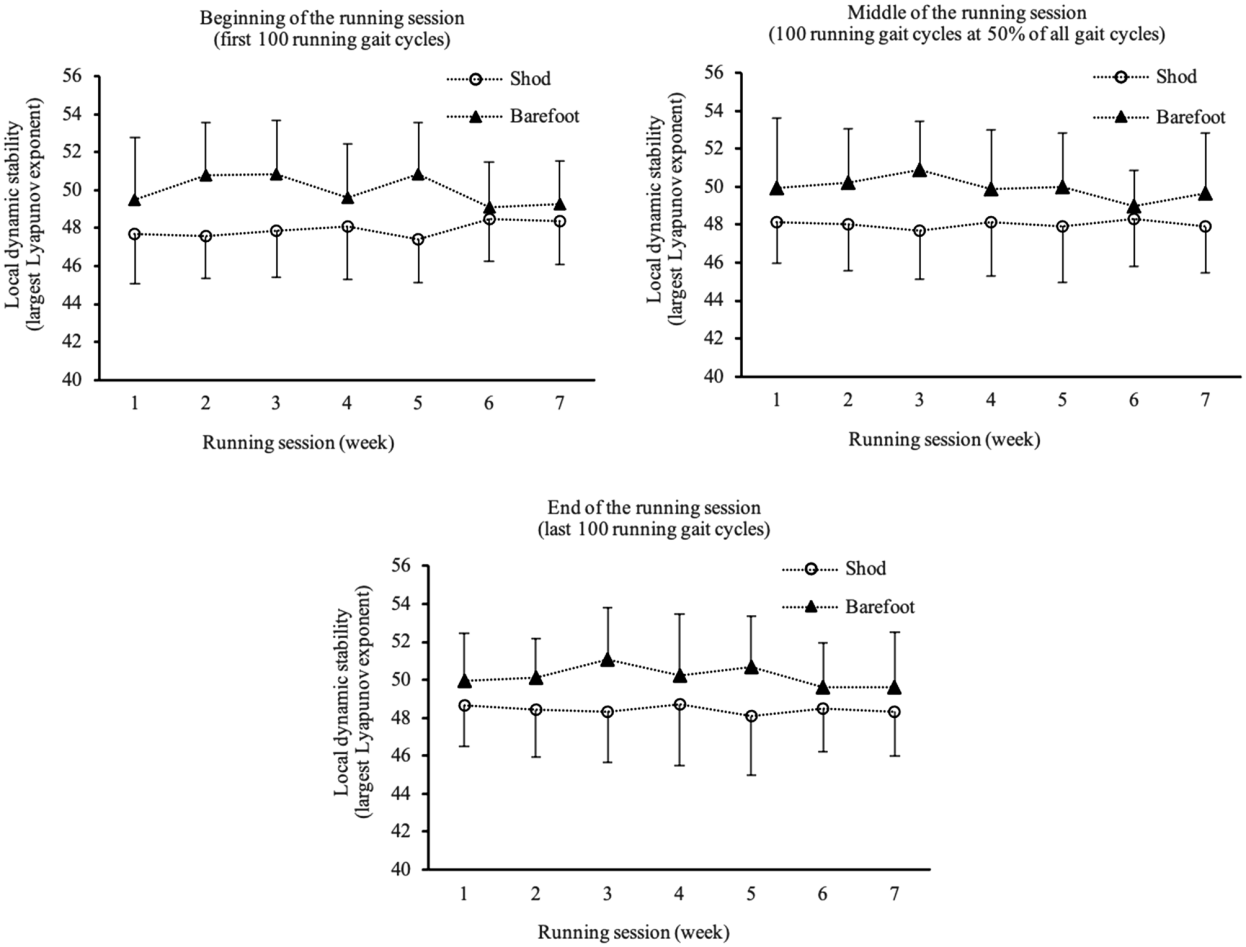
Local dynamic (running) stability (largest Lyapunov exponent) depicted for all seven running sessions for the beginning (3a), middle (3b) and end (3c) of the 15-min sessions.

Within the 15-min running sessions (from the beginning to the middle to the end of each session), running stability decreased (t_(__intrasession time__)_ = 2.5, *p* = 0.012, b = 0.579) in both groups (Table 2 and Fig. 3). There was a non-significant tendency that the decrease in running stability within a session was not as pronounced in the barefoot running group compared to shod running group (t_(intrasession time*group)_ = −1.9, *p* = 0.060, b = −0.612).

## Discussion

This randomized-controlled trial presents the results of a barefoot vs. shod running intervention over seven sessions on running stability. Running stability was lower in the barefoot running group compared to the shod running group, which was already existent in the first session. Furthermore, running stability did not change over the course of the intervention period, but was reduced over a single session of 15 min of running.

### Running barefoot versus shod affects running stability

The differences between the barefoot and shod group were existent from the first session and throughout the intervention period. A simple explanation could be a present random group effect as an alternative explanation to the interventional effect. This would be in line with a cross-over study by Frank et al.^40^, showing that cushioning properties of footwear seem to not acutely alter the local dynamic running stability. In contrast, with a similar cross-over design, it has been shown that running barefoot acutely affects the running stability^11^. When habitually shod runners switched to barefoot running, Ekizos, et al.^11^ described a lower running stability measured by local dynamic stability, which is in accordance with our results. While the cohort of habitually shod participants was similar, our study adds that the running stability was constantly lower over an 8-week/7-session running period in the barefoot group. While the underlying mechanisms of running stability are still mostly speculative, we expect an influence of the sensorimotor system on running stability^41,42^. Given the strength of a randomized controlled trial design and the finding of acute changes of footwear^11^, we conclude that barefoot running leads to reduced running stability compared to shod running in habitually shod runners.

Barefoot locomotion is thought to increase the sensory input (tactile and proprioceptive) and the non-habituated condition of barefoot running has been suggested to induce a less stable movement pattern^11,23,24,34,43^. Alternatively, footwear properties such as cushioning, flexibility and stability might also increase or decrease the running stability as it has been shown to influence several aspects of running biomechanics acutely and over time^6,21,44,45^. The acute changes in running stability and other biomechanics indicate that unfamiliar conditions such as a change of footwear are challenging for habitual running patterns. We expected that a repeated application to the new (barefoot) running condition would not only induce changes in foot strike patterns or ground reactions forces as seen in other studies^28,46–48^ but also in running stability. Another explanation could be that no full habituation was achieved over the 8 weeks with relatively few barefoot running (7 sessions of 15 min). It shall be emphasised that no running experience was required to qualify for this study and that all participants in this study were novice to barefoot running. There is no consensus what a habituated footwear status defines and there are currently no evidence-based recommendations on how to transition to (simulated) barefoot running^4,7^ as have been published for gait retraining protocols for injury prevention and rehabilitation^49^. Future investigations should focus on cohorts that are habituated for a longer period or habitually barefoot populations^50^.

### Running stability reduces during session

The running stability measured at the tibia decreased over the course of the 15 min of running in this study. In contrast, a recent study by Hoenig, et al.^12^ showed that running an exhaustive 5000 m run increased the running stability measured at the pelvis and thorax, but not at the foot. However, different time-delay as well as a different embedding dimension was used in our study. While the time was comparable for the competitive group (16–19 min), the difference between studies was that the velocity run in our study was non-exhaustive at 70% of the individual runners VO_2_ max velocity on a treadmill. It has been shown that acute fatigue influences dynamic stability of different motor tasks, while the underlying physiological mechanisms are not well understood^51–53^. Asgari, et al.^52^ interpreted that fatigue influenced the motor control and their ability to maintain repetitive dynamic tasks. It is has been shown that a cognitive component might also influence the ability to withstand small perturbations and increase local dynamic stability at least in walking^54^. Therefore, one could speculate that the monotonous task of running at a low speed on an indoor treadmill might decrease the awareness and attention of the participants on our study and decrease the cognitive resources used for the locomotors system to be kept stable.

### Strengths, limitations (Sources of bias) and generalizability

This was the first study to investigate repeated test condition effects of barefoot running on running stability measured by the largest Lyapunov exponent. While it can be determined from 3D motion capturing or IMU data, there is no consensus on the localisation of marker or IMU placement^12,16^. While Ekizos, et al.^16^ showed that marker clusters on the spine have high reliability to measure running stability with motion capture other studies have found different results depending on IMU location^12^. Further research is needed to investigate the impact of marker/IMU placement on running stability.

The population investigated consisted of active healthy adults that did not necessarily have a strong running background. Since it has been shown that the experience level of runners (recreational vs. competitive) has an effect on running stability^12^, caution needs no be taken when extrapolating the finding to other population, such as elite runners. As seen for biomechanics but also injuries, age and sex of the runners may also be of relevance for running stability^55,56^.

### Recommendations for further research

While some influencing factors on running stability (footwear, fatigue, running surface, running background) have been described in the literature, currently there is a need to better understand components contributing to this concept. Considering the trend of an intrasession decrease in the barefoot group future research should consider an individual perspective of adaptations, e.g. the relevance of responders and non-responders^47^. While the definition of running stability (ability of a dynamic system to compensate for small perturbations) is very precise, it is still to be determined whether a runner should strive for high or low running stability. Thus, practical (for running performance) and clinical implications (for running injuries) associated with an increased or decreased running stability should be in the focus of future research. Possible intervention strategies are still at an early stage of development. However, considering the wide availability of IMUs that can be worn on the body or in textiles as ‘wearables’^57–59^, the concept of running stability might be a promising field for future research.

## Conclusion

With good knowledge of the effects of footwear on running biomechanics, this study adds to the effects of footwear on the concept of running stability. In adults novice to barefoot running, running stability was lower in the barefoot running group when compared to the shod running group over the whole intervention period. This may be explained by long-term difficulties to adapt to the new running conditions and to develop a stable running pattern. Further studies are needed to explore if longer habituation periods to barefoot running are needed for improvements of running stability.

### Ethics approval and consent to participate

This study was approved by the institutional review board of the University of Hamburg (protocol number ID37) and was prospectively registered in the German Clinical Trial Register (DRKS00011073). Written informed consent was obtained prior to participation.

## Data Availability

All data generated or analysed during this study are included in this published article and its tables and figures, or is available upon request.
